# Lessons learned in preparing for and responding to the early stages of the COVID-19 pandemic: one simulation’s program experience adapting to the new normal

**DOI:** 10.1186/s41077-020-00128-y

**Published:** 2020-06-03

**Authors:** Ryan Brydges, Douglas M. Campbell, Lindsay Beavers, Nazanin Khodadoust, Paula Iantomasi, Kristen Sampson, Alberto Goffi, Filipe N. Caparica Santos, Andrew Petrosoniak

**Affiliations:** 1 Unity Health Toronto – Simulation Program, St. Michael’s Hospital, Unity Health Toronto, Toronto, Canada; 2 Department of Medicine, University of Toronto, Toronto, Canada; 3 Department of Paediatrics, University of Toronto, Toronto, Canada; 4 Department of Physical Therapy, University of Toronto, Toronto, Canada; 5 Department of Medicine, Division of Critical Care Medicine, St. Michael’s Hospital, Unity Health Toronto, Toronto, Canada; 6 Department of Medicine and Interdepartmental Division of Critical Care Medicine, University of Toronto, Toronto, Canada; 7 Department of Anesthesia, St. Michael’s Hospital, Unity Health Toronto, Toronto, Canada; 8 Department of Anesthesiology and Pain Medicine, University of Toronto, Toronto, Canada; 9 Department of Emergency Medicine, St. Michael’s Hospital, Toronto, Canada

**Keywords:** Healthcare simulation, Quality improvement and patient safety, Pandemic planning and response

## Abstract

Use of simulation to ensure an organization is ready for significant events, like COVID-19 pandemic, has shifted from a “backburner” training tool to a “first choice” strategy for ensuring individual, team, and system readiness. In this report, we summarize our simulation program’s response during the COVID-19 pandemic, including the associated challenges and lessons learned. We also reflect on anticipated changes within our program as we adapt to a “new normal” following this pandemic. We intend for this report to function as a guide for other simulation programs to consult as this COVID-19 crisis continues to unfold, and during future challenges within global healthcare systems. We argue that this pandemic has cemented simulation programs as fundamental for any healthcare organization interested in ensuring its workforce can adapt in times of crisis. With the right team and set of partners, we believe that sustained investments in a simulation program will amplify into immeasurable impacts across a healthcare system.

## Background

The coronavirus disease (COVID-19) pandemic has stressed our healthcare systems. Societal responses across the globe have pushed organizations and their employees to rely even more on various technologies to prepare for and respond to this crisis [1]. For instance, multiple groups have produced resources, like virtual cases [2] and tabletop simulations [3], to help frontline healthcare professionals (HCPs) and trainees address knowledge gaps [4], practice hands-on skills [5], and plan for patient care and systems challenges. Others have used their experience responding to COVID-19 outbreaks in China [6], Singapore [7], England [8], and in Norway, Denmark, and the UK [9] to share sage advice on the challenges and potential solutions when using both simulation and online learning modalities to ensure the safety of HCPs and their patients. Notably absent, however, are organizational- and program-level descriptions of how simulation programs have responded to their healthcare organization’s needs. We offer our lessons learned and insights gained from how the Unity Health Toronto – Simulation Program (UHT-SP) produced an educational, micro-systemic (i.e., processes within and between clinical units) [10], and macro-organizational response across our multi-institutional network.

One comprehensive account of simulation and technology-enhanced learning use during the COVID-19 pandemic noted that many Chinese organization’s views on simulation have shifted from it being a “backburner” training tool to a “first choice” technology for ensuring individual, team, and system readiness [6]. They note, however, that many recommendations for utilizing simulation (i.e., educational, hands-on focus) require time that most organizations, with their lagging response to the pandemic, do not have. To manage these challenges, the UHT-SP leadership team responded to multiple requests across our organization for simulation-based and technology-enhanced learning using two core principles: (i) functional task alignment (i.e., matching objectives to efficient technological solutions) [11, 12] and (ii) streamlined resource allocation (e.g., reusing and sustaining personal protective equipment (PPE)) [6]. In this report, we summarize our simulation program’s response during the COVID-19 pandemic, including the associated challenges and lessons learned. We also reflect on anticipated changes within our program as we adapt to a “new normal” following this pandemic.

## Our program and our response

### Our program’s baseline organizational structure

At UHT-SP, our organizational structure follows a format that many mature simulation programs may find familiar yet is bespoke to our unique hospital network. Our core team includes a director of technology-enabled education (NK), a director of research (RB), a medical director (DC), a program manager (LB), a translational simulation lead (AP), two certified simulation educators (PI, KS), and three simulation specialists. Our simulation educators, both trained and practicing as respiratory therapists, have a defined scope of practice that enables them to confidently work independently to teach physicians, nurses, and all other HCPs both at our simulation center and in in situ simulations (ISS) in the hospital. Alongside the simulation educators, clinician leads help tailor training to their unit’s needs, including nurse educators and physician leads in specialties like anesthesia (FCS) and critical care medicine (AG). To date, this structure has allowed us to nimbly respond to requests for education and to requests for studying organizational readiness [13] and latent safety threats in specific hospital units [14, 15].

### How the pandemic led us to reorganize our priorities

In January 2020, in anticipation of the COVID-19 clinical impact, our team prioritized unit-specific ISS over center-based simulation. Specifically, we helped the organization to develop and refine COVID-19-specific protocols for donning and doffing of PPE, and protected procedures (e.g., protected intubation, cardiac arrest). In February and March 2020, our team received overwhelming requests for on-the-job training from high-acuity units across two of our three hospital sites. We assigned dedicated sub-teams to conduct ISS that respected physical distancing guidelines, engaged HCPs in usability testing to test and refine policies, and helped us evaluate our educational approaches and enhance our curricular design. In these activities, we revised our standard operating procedure of having our UHT-SP team members rotate into and out of such initiatives. Instead, given the complexity and constantly changing protocols, we assigned members to sub-teams to address specific initiatives where they collaborated with speciality-based clinical simulation leads (where possible).

**Fig. 1:**
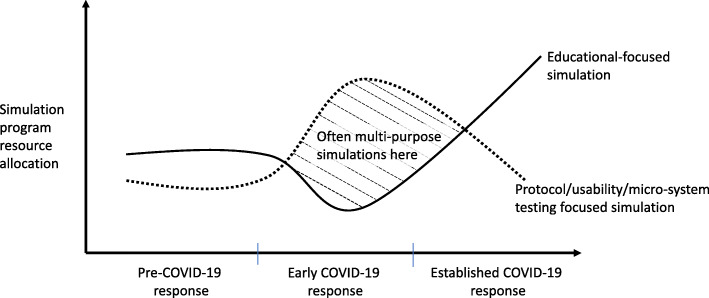
Representative timeline showing the UHT-SP’s transitioning efforts during the pre-COVID-19 period in 2020, as well as the early and later phases of the rise in cases in the Greater Toronto Area

As our organization finalized many of the COVID-19 protocols and checklists based on feedback from our simulation sessions, the workflows stabilized across our sites. This stability allowed us to revert back to our centralized model, aimed at upskilling HCPs in priority groups identified by our pandemic command centers. We focused on training professionals on units re-assigned to care for COVID-19 positive patients (e.g., our intensive care units—ICUs) and those high on the redeployment list (e.g., nurses and physicians expected to function as “delegate” clinicians in ICUs). Figure 1 provides a representative timeline of how our UHT-SP focus shifted over these early months.

Throughout these rapid-cycle transitions, requests for simulation support exceeded our resources. Based on our understanding of our organization, on the previous strategies our team has used successfully, and on each team member’s diverse foundational academic training, we used the following principles to allocate our efforts. Notably, we borrowed these principles from our previous design thinking and human factors work, behavioral psychology, and recommended approaches to change management.

i) Analysis of opportunity costs [16]: our resources were finite (i.e., supporting one initiative occurred at the expense of supporting another) so we implemented a prioritization scheme, and aimed to be as transparent as possible with stakeholders listed lower in that scheme.
ii) Prioritization [17]: we prioritized units that would be impacted early and significantly by COVID-19, together with individuals who could rapidly scale training throughout their teams (e.g., units with assigned and experienced simulation leads).
iii) Formation of legitimizing partnerships [17]: a key early enabler for our program was our ability to establish a strong relationship with the infection prevention and control (IPAC) office, which gave us credibility to act quickly and decisively across the organization.
iv) Standardization [18]: we developed “standard” lesson plans and scenario templates, based on daily updates to align with organizational guideline documents. In these efforts, we aimed to maintain consistency across multiple practice areas, while also allowing unit leaders to adapt to their local needs. This approach served as the catalyst to many efforts, especially on units without a dedicated simulation lead.
v) Adopting a multi-purpose lens [17]: given the sense of urgency, we often held targeted simulation sessions addressing multiple objectives, rather than our usual approach of addressing targeted objectives across multiple sessions.
vi) Iterative approach [13]*:* we adopted the perspective that perfection is the enemy of good, choosing to fail safely (i.e., run simulations, engage participants in structured feedback sessions, rapidly modify until the next version of a policy). This perspective served us well, given many of the hospital processes were undergoing constant beta testing as new evidence and opinion shaped decisions on a daily basis.
vii) Continuous brainstorming [13]: we stayed open to alternative ways of thinking about our colleagues’ options and activities, encouraging all team members to develop innovative strategies to function within the boundaries of new requirements (e.g., how to conduct simulations with physical distancing measures in place?).

## Taking the initiative to respond and our situated lessons learned

Our organization’s senior leadership recognized UHT-SP as a key change agent in Unity Health Toronto’s response to the multiple pandemic demands. For instance, our team has previously conducted several successful large-scale simulations aimed at testing clinical micro-system’s responsiveness to situations demanding high healthcare resources and patient surges, such as mock Code Silver [19], mock Code Oranges [20], and evaluation of a Family Information Support Centre model. Using the over-arching principle of functional task alignment [11]—aligning a simulation’s design, delivery, and function with the functional demands of the task at hand—we responded rapidly to the organization’s requests across a variety of initiatives. Below, we describe our response to three major questions asked of us by our organization’s senior leadership, and also provide further details about each initiative in Table 1. We shifted from an initial operational readiness and micro-systems focus [9], to an educational focus. The former involved experimentation and beta-testing policies and procedures, while the latter required that we ensure all HCPs and trainees had the minimum competence to practice safely when caring for COVID-19 positive patients.

**Table 1:** Description of the UHT-SP led initiatives in response to major requests from organizational senior leadership

Clinical target group/location	Overall objective(s)	Simulation and/or technology-enhanced modality	Impacts
**Organizational request: how can UHT-SP test new spaces and new models of care for COVID-19 patients?**			
All healthcare professionals screening COVID-19 patients.	To design a COVID-19 Assessment and Screening Centre with optimized physical spacing, staffing allocation, and patient flow	Iterative process using prototypes and mock-ups to guide construction of physical space, simulations with staff and standardized patients, iterative development of signage placement and design	Finalized data-informed protocols, signage, and workflows prior to the opening of the screening center
All critical care clinicians, as well as clinicians with potential to be redeployed to work in the ICU	To evaluate a proposed model of care, from primary care to team-based care, in anticipation of increased number of ICU patients and shortage of critical care trained clinicians	Videoconferencing to present the proposed model of care, multiple tabletop simulations using videoconference platform, in-person tabletop simulations for select groups, in-person in situ simulation in critical care setting	Derived themes from tabletop simulation discussions and synthesized into an executive summary about the model of care for professional practice teams and senior leadership Identified limitations of tabletop simulation led clinician participants to ask for the model to be piloted in actual ICUs with COVID-19 positive patients
**Organizational request: how can UHT-SP ensure our individuals and teams follow the safest clinical protocols and procedures?**			
Healthcare professionals and trainees working on various clinical units; each listed below with one example of each unit’s objectives.	To develop and refine hospital-based protocols in situ*,* and to feedforward information to leadership for command center decision-making	Rapid cycle in situ simulation scenarios focused on usability testing, identifying latent safety threats, and optimizing signage/visual aids; process was coupled with mock-ups and tabletop simulations	Identified and addressed gaps in new and pre-existing hospital policies and protocols Refined and finalized all policies and checklists/visual aids to guide further training to prepare for patient surges. Early simulation activities in the ICU sparked and cemented collaborations between UHT-SP, the IPAC team, and clinical units
Emergency department (ED)	Sample objective: to optimize the escalation protocol for transporting a COVID-19 positive patient from the ED to the ICU		
Intensive care unit (ICU)	Sample objective: to modify standard operating procedures to ensure they account for unique issues presented by COVID-19, including PPE use, novel specific COVID-19 equipment bundles, and “protected” procedures		
Operating room (ORs)	Sample objective: to translate the pre-existing PPE protocols developed by the IPAC team for non-OR areas to meet the needs of all perioperative staff, while maintaining IPAC established standards		
Labor and delivery (L&D) OR	Sample objective: to test and iteratively refine the policies associated with L&D team care for a laboring mom with a positive COVID-19 diagnosis		
Inpatient medical units	Sample objective: to implement protected code blue protocols established in the ICU on the acute care inpatient medical units, to determine how best to refine protocols in those settings		
Hospital morgue	Sample objective: to test and modify the protocols for transferring deceased COVID-19 positive patients from units to morgue and from morgue to funeral homes to inform the organization’s new expedited death response policy		
**Organizational request: how can UHT-SP ensure healthcare professionals have the minimal competence (and confidence) to practice safely?**			
Healthcare professionals, support staff, and trainees in the ED, ICU, ORs, L&D OR, inpatient medical units, and the morgue	To translate refined COVID-19 policies and protocols into training materials To train all healthcare professionals, repetitively where possible, to apply refined protocols to their general practices, as well as to specific procedures	In situ simulation scenarios in the early phase of protocol development; shifted to center-based simulation to run standardized scenarios for larger groups of healthcare professionals	Staff reported feeling less anxious, including an increased sense of safety and confidence following training. Practicing professionals, who typically view simulation as an educational tool for their trainees only, attended sessions in overwhelming numbers and their anecdotes suggest more extensive participation in future simulations.
First responders at all three sites	To ensure all first responders’ basic life support (BLS) skills meet the hospital network’s standard To expose learners to COVID-19 considerations, especially PPE use	Centralized curriculum, adapted to each site’s requirements in classroom or center-based setting; task trainers for protected BLS skills using “PPE buddy” approach	Upskilled approximately 180 participants Staff reported refresher helped reorganize their skills, and improved confidence they could stay safe and protected in their roles.
Registered nurses (RNs) across departments	To prepare non-critical care RNs to transition to work on COVID-19 ICUs via upskilling in, for example, aerosol-generating procedural skills	Center-based simulation, including part-task trainers, role play, and theater-based scenarios; train-the-trainer approach used to scale up the training from the original cohort to other nursing staff members	Completed training with 90 RNs, with most reporting reduced anxiety, increased confidence in providing safe care Simulation educators and trained RNs facilitators provided training for over 90 additional RN colleagues.

### Organizational request: how can UHT-SP test new spaces and new models of care for COVID-19 patients?

Our leadership chose UHT-SP to plan the COVID-19 Assessment and Screening Centre based on our team’s previous impacts ensuring organizational readiness in the emergency department (ED) [13], ICU, and other hospital units. We chose to conduct rapid cycle (i.e., over 2 days) simulations to develop the physical space allotments and overall flow of this critical piece of the organization’s COVID-19 arsenal. Our team employed usability-testing techniques (e.g., the iterative test-evaluate-design cycle [21]) and gradually increased the realism of processes following each cycle. Within each cycle, our team members encouraged participants to use think-aloud methods [22], allowing them to pause and express their perspectives around any confusions or uncertainties they experienced regarding patient and healthcare professional flow. Designing this space served as the starting point for our ongoing collaboration with the IPAC team, given the UHT-SP provided valuable data and feedback they used to finalize the physical layout, as well as the associated protocols for training staff to navigate the center query.

Another modality commonly used in systems-level disaster and emergency preparedness [23] and for identifying latent safety threats [24] is tabletop simulation [13]. Tabletop simulation scenarios typically involve participants using a physical blueprint of a space to “talk through” scenarios to identify potential deficiencies, inefficiencies, and barriers. We developed an innovative tabletop simulation in response to a request to evaluate a proposed model of care for COVID-19 dedicated ICUs. Our findings suggested the need for a significant change in practice for ICU nurses and physicians, who would shift their practice to manage groups of redeployed non-critical-care HCPs (e.g., physicians, nurses, respiratory and physical therapists) within a team-based care model. Given the constraints imposed by physical distancing measures, our team met on a videoconferencing platform to develop the scenario. We quickly realized that the technology would also ideally function as a “virtual tabletop” around which teams redeployed to the ICU could meet to evaluate the care model. Our team conducted three virtual tabletop simulations, producing a list of themes and potential challenges that informed leadership’s decision-making regarding the policies associated with the proposed model of care.

Once the organization’s senior leadership considered that these two bookends of care for COVID-19 patients had been established, from screening to increased ICU capacity, they and the UHT-SP leadership collectively decided to focus on the important gaps in between.

### Organizational request: how can UHT-SP ensure our individuals and teams follow the safest clinical protocols and procedures?

As others have outlined previously [25–31], many simulation modalities have been used to optimize the physical layout of patient rooms, how equipment is distributed within rooms, and how HCPs navigate those conditions [9]. Our initial ISS focused on activities marked as high acuity by our two priority units, the ED and the ICU. These activities included patient transport from the ED to the ICU and from the ICU to the OR, and protected code blue and intubation while in PPE. For these activities, we used principles initially developed in our work on the ICU, modified the activities to fit ED workflows, and aimed to use consistent clinical principles because many of the trained clinicians work in multiple units across our organization. These ISS also allowed us to develop and refine hospital-based protocols (e.g., a protected intubation checklist [32]), testing them and providing feedback and documentation for leadership approval. Once approved, we conducted ISS with other units to help them integrate, with some degree of domain specificity, these protocols into their workflows (e.g., a dedicated COVID-19 operating room (OR), the obstetrics OR, and the neonatal ICU).

As the UHT-SP met the early pandemic-related needs of our organization and the various clinical micro-systems, we shifted our priority back to an educational focus. Here, we aimed to ensure our HCPs received regular opportunities for just-in-time training in a variety of relevant skills.

### Organization’s request: how can UHT-SP ensure HCPs have the minimal competence (and confidence) to practice safely?

Simulation centers became ubiquitous globally largely based on evidence suggesting they offer a safe, ethical [33], and effective training environment for various educational needs across healthcare professions [34]. We conducted educationally oriented simulations, both ISS and center-based, to train various professionals in donning and doffing of PPE across many units (e.g., OR, ICU), first responders in basic life support skills refresher courses, clinicians (RNs, MDs, MD trainees, RTs) in care provision in high-risk environments involving aerosol-generating medical procedures (see Table 1). Given the time-sensitive nature of the training and our ongoing need to divide our team to meet the organization’s need, most of our education-focused sessions have not included formal assessments of competence. Instead, the current model involves leadership of relevant clinical units mandating that all frontline HCPs attend the training, actively participate in giving and receiving feedback, and return for repeated sessions if they desire.

## Our challenges and our mitigation strategies

In reviewing previous reports related to how simulation programs might respond to the COVID-19 pandemic, we found helpful tips about overcoming potential challenges. However, the descriptions focused narrowly on resource and time limitations [6], and on high-level collaboration, equipment, and coordination issues [9]. In addition to these important issues, which the UHT-SP also experienced, we describe below some of our key challenges. Our list expands upon previous reports to include how UHT-SP navigated personal and organizational politics, and how we managed daily shifts in what is considered consistent, high-quality, and accurate information to guide our practices. Several of the challenges listed are still ongoing.

### Key challenge: our organization’s requests exceeded our centralized UHT-SP team’s capacity

- Description: this represents an organization-level “stress” whereby demands exceed resources, a phenomenon that may be inevitable during a global pandemic. We continue to have difficulty keeping pace with the requests from our organization, and thus continue to implement our opportunity cost analysis and prioritization scheme.
- Mitigation strategy: despite forming our sub-teams, our centralized UHT-SP team’s capacity has been stretched. Thus, we have broadened our team to integrate “peripheral” simulation leads who previously worked with us occasionally into essential members of our team who we interact with daily.

### Key challenge: collating information that shifts daily to develop a unified, consistent response

- Description*:* the speed, uncertainty, and widespread impact of COVID-19 has formed a perfect storm of challenges related to unifying and collating information. Our ongoing ability to implement protocols endorsed by the organization into our practices has been substantially challenged by the near daily iterative changes that occur, as new information continuously shifts opinions.
- Mitigation strategy: our relationships with the IPAC team and with key clinical simulation leads, all strengthened by this pandemic, combined with our shift back to a *centralized training model* (Fig. 1), is helping our team become increasingly responsive to any subtle changes made to protocols, including how best to incorporate them into relevant curricula.

### Key challenge: organization’s request of urgent simulation-based education in clinical areas where existing policy and protocol gaps were unknown.

- Description: given an urgent need to rapidly prepare units to care for COVID-19 patients, leadership from some clinical areas (e.g., OR, ICU) requested simulation-based education for their staff. However, clinical leads, simulation leads, the IPAC team and UHT-SP learned quickly that existing protocols did not address context- and cultural- specific needs of those areas.
- Mitigation strategy*:* we identified existing gaps and area-specific needs during ongoing simulation sessions. During these sessions, we created, implemented, and refined context-specific protocols for those areas. Based on our pre-existing relationships with most unit simulation leads and educators, we used email and videoconferencing solutions to coordinate a collective response to all high-priority requests, to resolve any challenges, and to collaboratively navigate the various bureaucratic hurdles to achieve our objectives. UHT-SP provided input from simulation experiences that informed both the IPAC teams and the unit leadership’s decision-making processes, leading to the development of informed policies and protocols.

## How this changes us and our program

While we believe our program’s organizational structure and our pre-existing relationships with many of our partners enabled our robust response outlined above, we do anticipate several changes from the work spurred by the COVID-19 pandemic. Admittedly, some of what we report below amounts to an informed projection into the future. We categorize our insights according to expected changes in the organization, in individual clinical units and their related micro-systems, and in our program itself.

### Changes in our organization

**Fig. 2:**
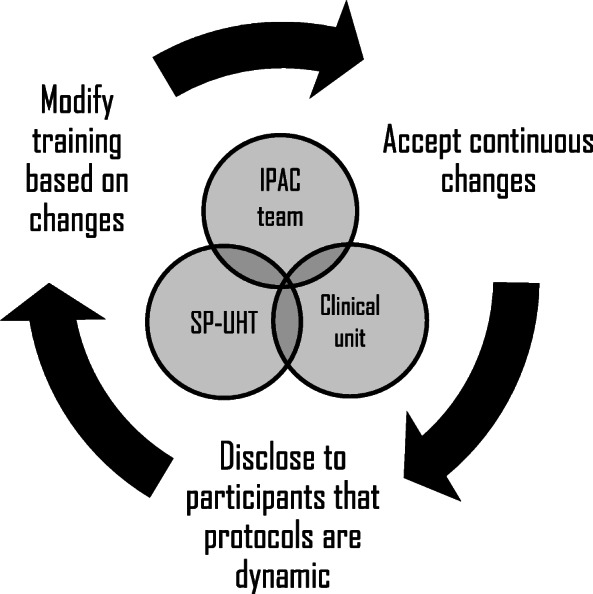
Depiction of the collaborative relationships between UHT-SP, the IPAC team, and the various clinical units that served as the foundation for ensuring our organization navigated the challenge of continuously shifting evidence and shifting protocols

Our experience aligns with the insights from China, which highlight that most organizations and simulation programs have done well simulating mass casualty scenarios, often at the expense of simulating the processes associated with infectious disease outbreaks [6]. As those authors recommend, and as we have experienced first-hand, a daily working relationship between UHT-SP and the IPAC team has been essential. Indeed, both groups functioned as links to different key stakeholders, which allowed our organization to do relatively well when navigating the challenge of shifting evidence and shifting protocols. Our specific lessons learned are summarized in Fig. 2:

(i) Accept that the current condition requires constant change,
(ii) Establish transparency to ensure everyone else—IPAC, educators, and learners—is aware of and acknowledges the period of constant change and uncertainty,
(iii) Openly amend training based on quality evidence, demarcating clearly what has and has not changed with each iteration, and
(iv) Loop back to having everyone accept that the most recent change likely will not be the last. The certainty of uncertainty in medicine becomes even more acute in a pandemic.

### Changes in our clinical units and the micro-systems interconnecting them

As we did in our own UHT-SP team, we observed great benefits when hospital portfolios and clinical units shifted their team structures from holistic to divisional. That is, by opting for a decentralized “divide and conquer” mindset, sub-teams became responsible for, and arguably made greater progress in, specific tasks [35]. For example, the Centre for Faculty Development redeployed an educator with simulation education experience to our team, who we assigned to work with nursing educators to plan and run the tabletop and ISS scenarios for testing the team-based model of care for COVID-19 ICUs. Despite such successes, we observed imperfections in how this model was applied across all facets of our organization and see this outcome as the basis to argue for a broad simulation scenario bank. Specifically, with lessons from this pandemic in mind, we can develop a broad set of “system shortage” scenarios that our team, our clinical micro-systems, and our organization can use to refine our proactive (and reduce our reactive) planning for future events of this magnitude.

Another key lesson to inform change relates to use of the term “standardized.” Across our experiences, leaders, educators, and learners expressed a strong desire for standard approaches, yet we often found that some customization within local contexts was required. We chose to apply standardized principles that were adjusted to reflect domain specificity within each unit’s culture, patient population, and equipment availability. To illustrate this lesson, we have yet to confirm a universally “standard” approach to PPE donning and doffing because unique needs—both evidence-based and political/bureaucratic—have arisen regularly within different clinical units (e.g., OR vs. ICU), hospital sites (e.g., urban vs. community), and clinical specialties (e.g., anesthesia vs. general medicine vs. respiratory therapy). Hence, while “standardized” has become immutable in the lexicon of academic healthcare, and is often appropriately associated with safety and reliability, we argue that strictly applying the term may set unrealistic expectations, especially in times of uncertainty where the need to accept constant change tends to rule each day (see above).

### Changes in the UHT-SP’s practices

Firstly, we deepened our relationships with our pre-existing clinical simulation leads during this response to the pandemic. A key change going forward for UHT-SP involves identifying and formally appointing interprofessional simulation leads across our clinical units and across our three sites. Doing so will meet three objectives: (i) to build a larger cadre of people to enhance our capability to rapidly respond to future challenges, (ii) to establish UHT-SP as a go-to program for planning and evaluating new spaces and protocols, and (iii) to better integrate UHT-SP team members into the *ongoing* decision-making processes and working groups on clinical units. To accomplish these objectives, we will need to develop a recruitment strategy, refine our train the trainer programs, and coordinate regularly scheduled events to build this community.

Secondly, we have learned about the impacts of infectious disease outbreaks on our program and the need to change accordingly. Examples include enhancing the sanitation standards for disinfecting simulators and simulation rooms between uses, and judiciously using and conserving PPE in all of our training and assessment activities (e.g., using expired and damaged equipment, applying disposable products, like cling wrap, to face shields to facilitate reuse).

Thirdly, we re-considered how we work together with our learners and partners. Our development of the “virtual tabletop simulation” respects physical distancing concerns and may eventually represent a more efficient version of the modality. Our program will investigate the merits and challenges of video-recording certain types of simulation (e.g., testing of protocols, evaluation of physical layouts) that previously required bringing together groups of participants and observers. Rather than meeting in person, the resulting video could be shown over videoconferencing, to permit a virtual debrief with relevant stakeholders. Developing a bank of such videos could also represent an educational repository for various learner groups. As Benjamin Franklin said, “out of adversity comes opportunity,” and the pandemic now imposes the need for us to develop new protocols for deciding between virtual and physical versions of simulations that we previously delivered in person.

## Conclusion

When used with purpose by trained professionals, simulation goes well beyond a static modality, bound to a singular center. While we acknowledge that this report serves to showcase the efforts put forth by our UHT-SP team, we intend for it to function as a guide that other simulation programs can consult as this COVID-19 crisis continues to unfold, and during future challenges to global healthcare systems. We argue that this pandemic has cemented simulation programs as fundamental for any healthcare organization interested in ensuring its workforce can adapt in times of crisis. Simulation has become as much a tool for organizational learning, as it has been for individual and team learning. With the right team and set of partners, we believe that sustained investments in a simulation program will amplify into immeasurable impacts across a healthcare system.

## Data Availability

We did not collect or report data for the purposes of this report.

## Abbreviations

UHT-SP: Unity Health Toronto – Simulation Program
HCP: Healthcare professional
PPE: Personal protective equipment
ICU: Intensive care unit
ISS: In situ simulation
ED: Emergency department
IPAC: Infection prevention and control
RN: Registered nurse
MD: Medical doctor
RT: Respiratory therapist
